# Male and female hypertrophic rat cardiac myocyte functional responses to ischemic stress and β-adrenergic challenge are different

**DOI:** 10.1186/s13293-016-0084-8

**Published:** 2016-07-07

**Authors:** James R. Bell, Claire L. Curl, Tristan W. Harding, Martin Vila Petroff, Stephen B. Harrap, Lea M. D. Delbridge

**Affiliations:** Department of Physiology, University of Melbourne, Melbourne, Victoria Australia; Centro de Investigaciones Cardiovasculares, Centro Cientifico Tecnologico La Plata, Facultad de Ciencias Medicas, Universidad Nacional de La Plata, La Plata, Argentina; Cardiac Phenomics Laboratory, Department of Physiology, University of Melbourne, Melbourne, Victoria 3010 Australia

**Keywords:** Cardiomyocyte, Sex/gender, Cardiac hypertrophy, Ischemia/reperfusion, Stress response

## Abstract

**Background:**

Cardiac hypertrophy is the most potent cardiovascular risk factor after age, and relative mortality risk linked with cardiac hypertrophy is greater in women. Ischemic heart disease is the most common form of cardiovascular pathology for both men and women, yet significant differences in incidence and outcomes exist between the sexes. Cardiac hypertrophy and ischemia are frequently occurring dual pathologies. Whether the cellular (cardiomyocyte) mechanisms underlying myocardial damage differ in women and men remains to be determined. In this study, utilizing an in vitro experimental approach, our goal was to examine the proposition that responses of male/female cardiomyocytes to ischemic (and adrenergic) stress may be differentially modulated by the presence of pre-existing cardiac hypertrophy.

**Methods:**

We used a novel normotensive custom-derived hypertrophic heart rat (HHR; vs control strain normal heart rat (NHR)). Cardiomyocyte morphologic and electromechanical functional studies were performed using microfluorimetric techniques involving simulated ischemia/reperfusion protocols.

**Results:**

HHR females exhibited pronounced cardiac/cardiomyocyte enlargement, equivalent to males. Under basal conditions, a lower twitch amplitude in female myocytes was prominent in normal but not in hypertrophic myocytes. The cardiomyocyte Ca^2+^ responses to β-adrenergic challenge differed in hypertrophic male and female cardiomyocytes, with the accentuated response in males abrogated in females—even while contractile responses were similar. In simulated ischemia, a marked and selective elevation of end-ischemia Ca^2+^ in normal female myocytes was completely suppressed in hypertrophic female myocytes—even though all groups demonstrated similar shifts in myocyte contractile performance. After 30 min of simulated reperfusion, the Ca^2+^ desensitization characterizing the male response was distinctively absent in female cardiomyocytes.

**Conclusions:**

Our data demonstrate that cardiac hypertrophy produces dramatically different basal and stress-induced pathophenotypes in female- and male-origin cardiomyocytes. The lower Ca^2+^ operational status characteristic of female (vs male) cardiomyocytes comprising normal hearts is not exhibited by myocytes of hypertrophic hearts. After ischemia/reperfusion, availability of activator Ca^2+^ is suppressed in female hypertrophic myocytes, whereas sensitivity to Ca^2+^ is blunted in male hypertrophic myocytes. These findings demonstrate that selective intervention strategies should be pursued to optimize post-ischemic electromechanical support for male and female hypertrophic hearts.

**Electronic supplementary material:**

The online version of this article (doi:10.1186/s13293-016-0084-8) contains supplementary material, which is available to authorized users.

## Background

For both women and men, cardiovascular disease is the leading cause of death and disability [1, 2]. Cardiac hypertrophy is the most potent cardiovascular risk factor after age [3], and importantly, relative mortality risk linked with cardiac hypertrophy is greater in women [4]. There is a growing clinical recognition that myocardial cardiac stress responses, including neurohumoral activation, exhibit inherent sex difference [5].

Ischemic heart disease is the most common form of cardiovascular pathology for men and women [6]. Ischemic events occur earlier in men, and ischemia-related arrhythmia and sudden cardiac death incidence are higher in men. In contrast, while younger women have a lower risk of ischemic heart disease, in the event of a myocardial infarct, they experience higher mortality and rate of heart failure, even though reperfusion status is equivalent [7, 8]. Women with ischemic heart disease have higher rates of hospitalization than men [9]. The essential question of whether the mechanisms underlying ischemic heart disease in women differ from men remains unanswered [10]—and the cognate issue of whether ischemia coincident with hypertrophic comorbidity has differing gender aetiology and outcome has not been addressed.

The importance of achieving a more detailed understanding of basic mechanisms of sex difference in pathophysiologic processes, which have particular relevance to cardiovascular disease and health demography, has been emphasized [11]. The value of sex-inclusive quality pre-clinical data to inform large-scale clinical studies has also been recently highlighted in the context of unexpected findings delivered by the RELAX trial—which sildenafil (a phosphodiesterase 5 inhibitor) did not show a beneficial effect in the treatment of heart failure with preserved ejection fraction [12]. In retrospect, it became apparent that this outcome might have been predicted if the rationale for the trial had not been reliant on data from male-only human and animal studies [13, 14].

Experimentally, important insights have been gained in relation to sex-specific differentials in the impacts of cardiac hypertrophy and of exposure to ischemic insult—yet still, knowledge is lacking [15, 16]. The extent of hypertrophic remodeling in female rodents in response to loading stress is generally attenuated, yet exogenous oestrogen and oestrogen receptor (oestrogen receptor α (ERα)) agonists have been shown to increase mortality in mice subjected to aortic constriction or to infarction [17–19]. Generally, female hearts are less susceptible to post-ischemic contractile dysfunction, findings which primarily derive from experiments involving animal models where function is not compromised by pre-existing pathologies [15, 20–22]. We have previously established that the intrinsic resistance of isolated female hearts to ischemic insult is abrogated in a setting of primary cardiac hypertrophy [23].

Most fundamentally, protection against hypertrophy and ischemia-driven arrhythmogenesis and contractile dysfunction is determined by cardiomyocyte Ca^2+^ regulatory status. Whilst limited sex-specific information is available at the myocyte level, in vitro findings suggest differential female and male contractile/Ca^2+^ handling characteristics in simulated ischemia and also sex differences in the responsiveness of myocytes to manipulation of adrenergic activation [24, 25]. Specifically, it is reported that during simulated ischemia, only female cardiomyocytes exhibit a reduction in Ca^2+^ transient amplitude (vs non-ischemic control) and that in reperfusion, female cardiomyocytes are hyper-contractile (vs males) and more likely to survive the ischemia/reperfusion challenge [24]. Sex differences in adrenergic responses apparently reflect reduced cyclic adenosine monophosphate (cAMP) levels in female myocytes (vs male), contributing to lower Ca^2+^ levels and internal Ca^2+^ store flux responses [25]. Importantly, no previous cardiomyocyte functional studies have interrogated the role of pre-existing hypertrophic pathology in determining the sex-specific responses to ischemic injury—a highly relevant clinical scenario of comorbidity [26, 27].

In this study, utilizing an in vitro experimental approach, our goal was to examine the proposition that responses of male and female cardiomyocytes to stress stimuli (β-adrenergic activation and simulated ischemia/reperfusion insult) may be differentially affected by the presence of pre-existing cardiac hypertrophy. Using a purpose-derived rat model of normotensive cardiac hypertrophy, the hypertrophic heart rat (HHR), a range of cardiomyocyte morphologic and electromechanical functional studies was performed using microfluorimetric techniques. We have previously reported premature mortality in the HHR (vs normal heart rat (NHR)), with ex vivo HHR exhibiting normal systolic function and an increased vulnerability to ischemia/reperfusion dysfunction/injury [23, 28]. The present study now demonstrates that cardiac hypertrophy produces dramatically different pathophenotypes in female- and male-origin cardiomyocytes exposed to ischemia. These findings suggest implications for sex-specific refinement of reperfusion therapeutic interventions.

## Methods

### Animals

Animals were obtained from established colonies of the HHR and the control NHR, maintained at the Biological Research Facility at the University of Melbourne, Australia, and derived as reported [29]. Briefly, these rats originated from the F2 progeny of a cross between the Fischer 344 and spontaneously hypertensive rat (SHR). Normotensive offspring were selected for either enlarged or normal heart size by echocardiography over successive generations to achieve genetic stability. Telemetry, tail-cuff plethysmography and direct intra-arterial recording methods have been used to determine the normotensive status of these animals [29]. Experiments were conducted and animals handled in the manner specified by the NHMRC/CSIRO/ACC Australian Code of Practice for the Care and Use of Animals for Scientific Purposes (1997) and the EU Directive 2010/63/EU for animal experiments, with approval and oversight by the University of Melbourne Animal Ethics Committee.

### Cardiomyocyte isolation procedure

Ventricular myocytes were isolated from NHR and HHR rats, as described [30]. Briefly, animals were weighed, killed by decapitation under deep halothane anaesthesia, hearts were excised, trimmed, buffer washed and blotted prior to taking weight measurement by tare. Hearts were retrogradely perfused with bicarbonate-buffered Ca^2+^-free Krebs (in millimolars: 118 NaCl, 4.8 KCl, 1.2 KH_2_PO_4_, 1.2 MgCl_2_, 25 NaHCO_3_, 11 d-glucose) at 73 mmHg for 3 min at 37 °C. Cardiomyocytes were then perfused with type II collagenase (50 mg/ml, 295 U/mg; Worthington Biochemical Corporation, NJ, USA) to enable heart digestion. The heart was removed from the cannula, and the ventricular cardiomyocytes dispersed in Krebs/collagenase solution (in millimolars: 146.2 NaCl, 4.7 KCl, 0.4 NaH_2_PO_4_-H_2_O, 1.1 MgSO_4_-7H_2_O, 10 (4-(2-hydroxyethyl)-1-piperazineethanesulfonic acid (HEPES), 11 d-glucose) supplemented with trypsin inhibitor (25 μg/ml; Sigma-Aldrich, MO, USA) to inhibit residual collagenase activity. For each heart, 100 rod-shaped and regularly striated cardiomyocytes were selected at random for length and width measurement at ×400 magnification using an inverted light microscope and calibrated eye piece as previously described [31]. Cardiomyocyte volume was derived from the product of cell length and width, multiplied by 7.59 × 10^−3^ pl/μm^2^ [32].

### Cardiomyocyte Ca^2+^ handling and twitch analysis

Isolated myocyte Ca^2+^ levels and shortening performance were measured [30]. Cell suspensions were incubated with the Ca^2+^ fluorescent dye, fura2-AM (2.5 μM, 20 min, 25 °C; Invitrogen). Myocytes were superfused with 2 mM Ca^2+^-HEPES buffer (in millimolars: 146.2 NaCl, 4.7 KCl, 0.4 NaH_2_PO_4_H_2_O, 1.1 MgSO_4_7H_2_O, 10 HEPES, 11 glucose, pH 7.4) and stimulated to establish steady state contractile performance at 4 Hz (5 min, 37 °C). Cardiomyocyte twitch properties were assessed by video-based edge detection (IonOptix, MA, USA). The indices used to describe twitch cycle were twitch amplitude normalized to diastolic cell length (twitch amplitude percentage) and maximal rate of shortening and lengthening (mrs and mrl, respectively; μm/s, normalized to diastolic cell length). Myocyte Ca^2+^ signals were measured by microfluorimetry (360:380 nm fluorescence ratio, 1000 Hz sampling; IonOptix, MA, USA) [30]. Fluorescence signals were corrected for background. The indices used to describe the Ca^2+^ transient were amplitude (*F*_360:380_) and the time constant of decay (tau, ms; time constant measured from curve fitted from 10 % below the peak level to baseline of transient). Cardiomyocyte twitch and Ca^2+^ fluorescent ratio properties were measured off-line using IonWizard (IonOptix) and were determined by average of ten steady state transients for each myocyte. Ca^2+^-shortening phase loops were constructed for individual activation cycles by plotting Ca^2+^ level vs myocyte shortening throughout the shortening and lengthening phases of the cycle. During the relaxation (i.e. lengthening) phase, the Ca^2+^ level at half (50 %) relaxation may be interpreted as an index of myofilament Ca^2+^ sensitivity [33].

### Adrenergic challenge protocol

To assess the response of male and female NHR and HHR cardiomyocytes to an adrenergic challenge, myocytes stabilized for 5 min in 2 mM Ca^2+^-HEPES buffer were superfused with 10 nM isoproterenol for 5 min. Previous dose range and time-course studies confirmed this was an optimal time point to allow a maximal shortening and amplitude Ca^2+^ response to reach a new steady state [34].

### Simulated ischemia/reperfusion protocol

To assess the response of male and female NHR and HHR cardiomyocytes to an ischemia and reperfusion challenge, cardiomyocytes were superfused with a modified HEPES buffer (in millimolars: 136 NaCl, 8 KCl, 0.4 NaH_2_PO_4_-H_2_O, 1.1 MgSO_4_-7H_2_O, 2.0 CaCl_2_, 10 HEPES, 10 lactate, pH 6.8, 100 % N_2_ gas saturation) for 20 min to simulate conditions associated with ischemia, including hypoxia, hypercapnia, acidosis, substrate deprivation and lactate accumulation [30, 35, 36]. At the end of the 20-min superfusion with the simulated ischemia solution, the perfusate was switched back to the basal 2-mM Ca^2+^-HEPES buffer for a further 30 min. Cardiomyocytes were electrically stimulated (4 Hz) throughout the ischemia/reperfusion protocol.

### Statistics

Data are presented as mean ± SEM. Differences between groups were assessed by two-way ANOVA and with repeated measures as appropriate. Myocyte survival curves were assessed by log-rank Mantel-Cox test. Data were considered significant at *p* < 0.05. All statistical calculations were performed using SPSS V.22.0 (IBM Corp, NY, USA). Statistical annotation used throughout the study to designate significance level: #, strain effect; *, sex effect; †, sex-strain interaction. Post hoc analysis was performed on data in which a sex-strain interaction was evident, with significant differences in post hoc analysis represented in the figures (§, *p* < 0.05, male vs female of same strain) directly above the relevant figure bar or data point.

## Results

### Cardiac and cardiomyocyte hypertrophy in male and female HHR

Female body and heart weights were significantly lower than strain and age-matched NHR and HHR values (Table 1). Heart weight and cardiomyocyte dimensions were smaller in female NHR (Table 1 and Fig. 1, respectively), yet cardiac weight index (CWI; g/kg) was greater compared with male NHR. In HHR, cardiac and cardiomyocyte hypertrophy was most pronounced in female with augmented CWI associated with more substantial myocyte enlargement in width (and consequently volume) dimension (Table 1 and Fig. 1, †*p* < 0.05; Fig. 1b–d). These data confirm a robust hypertrophic phenotype in female and male HHR.

**Table 1:** Age and body/heart weights of male and female NHR and HHR

Parameter	NHR		HHR	
	Male	Female	Male	Female
Age (weeks)	14.6 ± 0.7 (15)	13.3 ± 0.3 (13)	14.8 ± 1.0 (14)	13.7 ± 0.6 (13)
Body weight (g)	352 ± 7.9 (15)	180 ± 5.5^#^ (13)	299 ± 10.2^##^ (14)	171 ± 4.7^##^ (13)
Heart weight (mg)	1410 ± 28.5 (15)	882 ± 27.7^#^ (13)	1514 ± 85.8 (14)	1202 ± 69.2^#,##^ (13)
CWI (mg/g)	4.02 ± 0.1 (15)	4.91 ± 0.1^#^ (13)	5.12 ± 0.3^##^ (14)	7.04 ± 0.4^#, ##^ (13)

#sex *p* < 0.05; ##strain *p* < 0.05, mean ± SEM, *n* = hearts or animals in brackets for each group

**Fig. 1:**
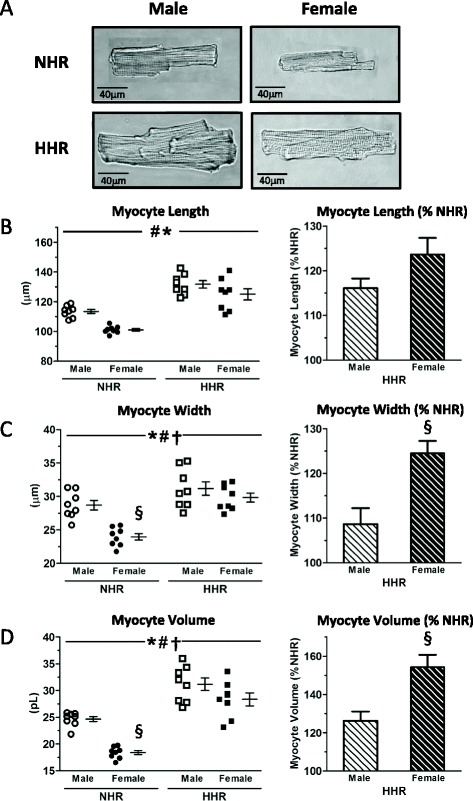
Cardiomyocyte hypertrophy in male and female HHR. **a** Representative images of cardiomyocytes from male and female NHR and HHR hearts. **b**–**d** Augmented CWI associated with more substantial myocyte enlargement in width (and consequently volume) dimension in female vs male HHR (*sex *p* < 0.05; #strain *p* < 0.05; †sex-strain interaction; §post hoc male vs female *p* < 0.05, mean ± SEM. Each data point mean of *n* = 50 cells from each of eight hearts/group, total 400 cells/gp)

### Cardiomyocyte basal performance—sex differences suppressed with hypertrophy

**Fig. 2:**
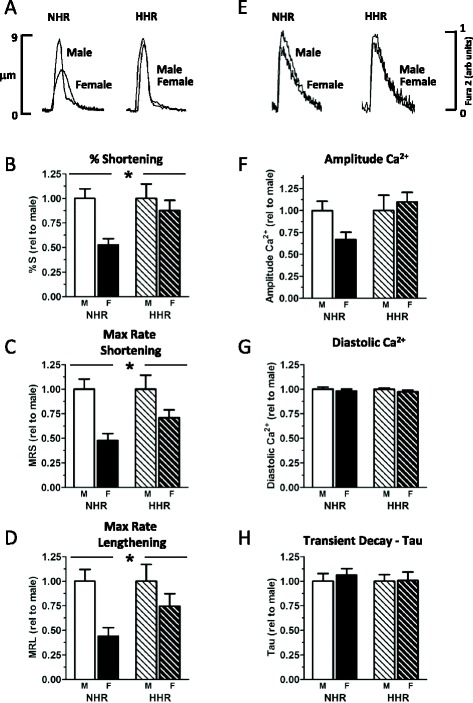
Basal sex differences in cardiomyocyte performance suppressed with hypertrophy. **a** Representative records of myocyte shortening in male and female NHR and HHR cardiomyocytes. **b**–**d** Basal twitch amplitude (% shortening), maximum rate of shortening/lengthening are lower in female cardiomyocytes compared with males—an effect that is accentuated in NHR compared with HHR. **e** No sex difference was observed in Ca^2+^ transients, though a trend for lower Ca^2+^ transient amplitude was evident in female NHR cardiomyocytes (**f**). No sex difference was observed in cardiomyocyte diastolic Ca^2+^ levels or the time constant of Ca^2+^ transient decay (**g**, **h**). (*sex *p* < 0.05; *p* < 0.05; mean ± SEM; *n* = 8–13 cells from four to five hearts/group)

Basal NHR cardiomyocyte contractile performance was lower in female cardiomyocytes compared with males (Fig. *2*a–d, Additional file 1: Table S1 *p < 0.05). This lower female contractility was marked in normal female myocytes (approximately 50 % lower of the percentage shortening values vs male NHR), though time to peak shortening was particularly slow in female HHR cardiomyocytes (Additional file 1: Table S1). Lower contractility in normal female myocytes was associated with a trend for lower Ca^2+^ transient amplitude and systolic Ca^2+^ (Fig. 2e, f, Additional file 1: Table S1). No sex difference was observed in NHR cardiomyocyte diastolic Ca^2+^ levels or the time constant of Ca^2+^ transient decay (Fig. 2g, h). Lower contractility was not apparent in female HHR cardiomyocytes relative to male (Fig. 2b). As for NHR, overall Ca^2+^ levels did not differ between male and female HHR myocytes (Fig. 2e–h). These data show that basal contractile performance in non-hypertrophic male and female cardiomyocytes is fundamentally different, but this sex differential is lost when myocytes exhibit pathological hypertrophy.

### Augmented HHR Ca^2+^ activator response to β-adrenoceptor stimulation in male cardiomyocytes absent in female cardiomyocytes

**Fig. 3:**
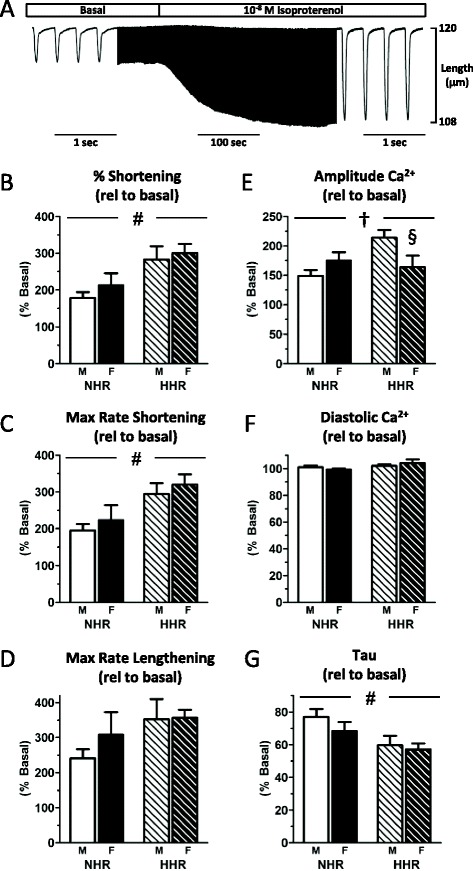
β-Adrenoceptor stimulation Ca^2+^response blunted in hypertrophic female HHR cardiomyocytes. **a** Representative cardiomyocyte shortening trace (central portion time compressed) showing isoproterenol (10 nM) exposure-elicited response of rapid and sustained increase in cell twitch. **b**–**d** The extent of increase in shortening and maximum rate of shortening was similar in male and female cardiomyocytes, but more pronounced in the presence of pathological hypertrophy. Rate of lengthening was unchanged. **e** The extent of isoproterenol-induced increase in Ca^2+^ transient amplitude was selectively blunted in female HHR myocytes. **f** Diastolic Ca^2+^ levels were stable with isoproterenol challenge in all treatment groups, whilst tau (**g**) was significantly faster in HHR of both sexes (#strain *p* < 0.05; †sex-strain interaction; §post hoc male vs female *p* < 0.05, mean ± SEM, *n* = 9–11 cells from four to five hearts/group)

β-Adrenoceptor agonist challenge in vitro elicited a substantial increase in cardiomyocyte contractility (Fig. 3a, Additional file 2: Table S2). The magnitude of this response was similar in male and female cardiomyocytes, but more pronounced in the presence of hypertrophy (Fig. 3b–d, M NHR 178.9 ± 16.1 %, F NHR 213.5 ± 32.1 %, M HHR 282.9 ± 36.3 %, F NHR 300.7 ± 25.2 %, #*p* < 0.05). Sex differences in the Ca^2+^ response to β-adrenoceptor stimulation were evident. The extent of isoproterenol-induced increase in Ca^2+^ transient amplitude was selectively blunted in female HHR myocytes compared with males (Fig. 3e, †*p* < 0.05). This sex-specific response was not observed in non-hypertrophic (NHR) myocytes. Diastolic Ca^2+^ levels were stable with isoproterenol challenge in all treatment groups (Fig. 3f). A more rapid transient decay (decrease in tau) was evident in hypertrophic male and female cardiomyocytes, consistent with a trend for increased maximum rate of lengthening (Fig. 3d, g). These data demonstrate that the Ca^2+^ response to β-adrenergic stimulation is different in male and female hypertrophic cardiomyocytes—the accentuated response in HHR male myocytes is not achieved in female myocytes.

### Selective Ca^2+^ elevation in female myocytes at end ischemia abrogated with hypertrophy

**Fig. 4:**
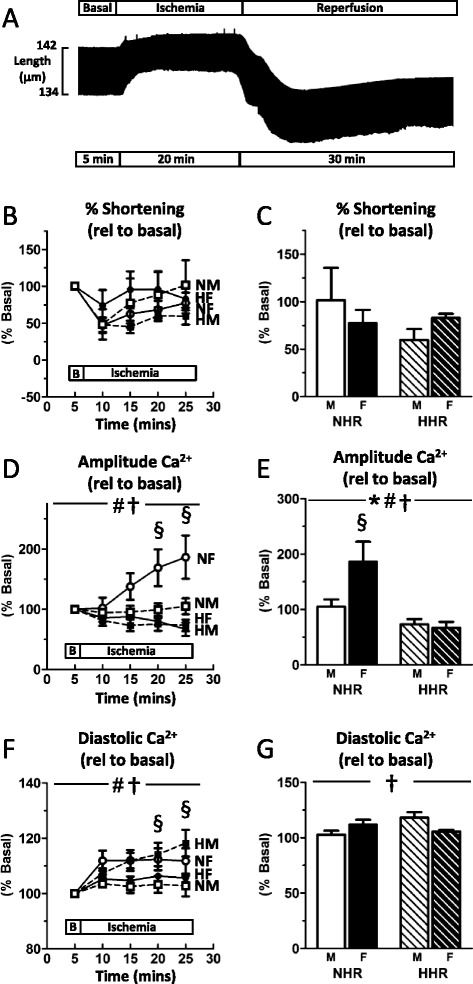
Selective Ca^2+^ elevation in female myocytes at end ischemia is abrogated with hypertrophy. **a** Representative cardiomyocyte shortening record shows modulated diastolic length and twitch amplitude in simulated ischemia and reperfusion (time compressed). **b** All groups show a similar pattern of shortening response to simulated ischemia. **c** No significant difference between groups was detected in the extent of change in twitch reduction at end ischemia. **d** Ca^2+^ transient responses during the time-course of simulated ischemia. **e** Female NHR cardiomyocytes exhibited a substantial increase in amplitude Ca^2+^ at the end of ischemia which was not evident in female hypertrophic myocytes. **f** Diastolic Ca^2+^ increased throughout ischemia in male HHR cardiomyoctes. **g** At end ischemia, increased diastolic Ca^2+^ in male HHR was absent in female HHR myocytes. (*sex *p* < 0.05; #strain *p* < 0.05; †sex-strain interaction; §post hoc male vs female *p* < 0.05; mean ± SEM; *n* = 7–14 cells from 6 to 11 hearts/group)

Myocyte contractile performance and Ca^2+^ levels were tracked throughout the time-course of a simulated ischemia/reperfusion challenge (sample time-compressed record, Fig. 4a, Additional file 2: Table S2). At the commencement of ischemia, all cell groups demonstrated an abrupt decrease in twitch amplitude and a small increase in resting length. This reduction in twitch amplitude reached a plateau within the first 5 min of simulated ischemia (Fig. 4b), with no significant difference between groups in the extent of twitch amplitude reduction at end ischemia (Fig. 4c). Ca^2+^ transient responses to simulated ischemia were very different in male and female cardiomyocytes (Fig. 4d, †*p* < 0.05). In contrast to males, female NHR cardiomyocytes exhibited a substantial increase in amplitude Ca^2+^ at the end of ischemia (relative to pre-ischemic basal; M NHR 104.8 ± 13.3 %, F NHR 186.2 ± 35.9 %, *p < 0.05), which was not associated with a concomitant increase in shortening (Fig. 4e). This marked and selective elevation of end-ischemia Ca^2+^ in female NHR was totally abrogated with hypertrophy (HHR, Fig. 4e, M HHR 73.1 ± 9.5, F HHR 66.8 ± 10.9 %). Despite a significantly lower Ca^2+^ signal in hypertrophic cardiomyocytes (Fig. 4e, #*p* < 0.05), the shortening responses between male and female HHR were not different (Fig. 4c, *p* = ns). A sex-strain interaction was evident for diastolic Ca^2+^ during simulated ischemia (Fig. 4f, g). An increase in diastolic Ca^2+^ in male HHR cardiomyocytes was not evident in female HHR, which remained at near-basal levels of diastolic Ca^2+^ (Fig. 4g). This contrasted with NHR myocytes, with a trend for greater diastolic Ca^2+^ at the end of simulated ischemia compared with males.

### Myofilament Ca^2+^ desensitization in reperfused hypertrophic male myocytes is absent in females

**Fig. 5:**
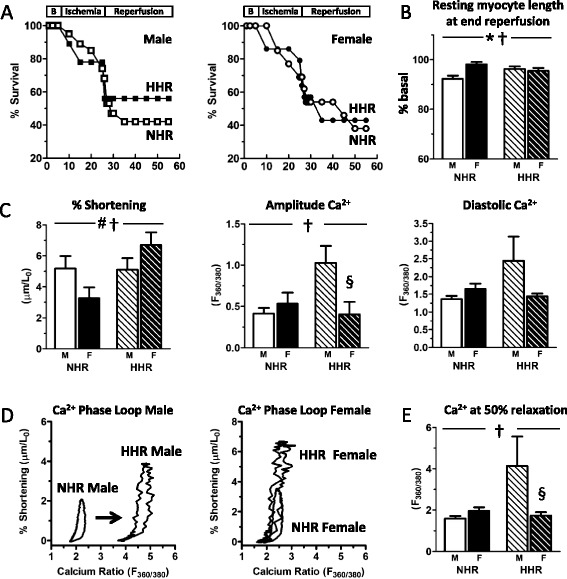
Myofilament Ca^2+^ desensitization in hypertrophic male myocytes at the end of reperfusion is absent in females. **a** Cardiomyocyte survival plots throughout simulated ischemia and reperfusion. **b** Resting myocyte length decreased to a greater extent in male NHR cardiomyocytes compared with females, with this sex difference absent in the HHR. **c** At end reperfusion, male HHR cardiomyocytes maintain contractility yet exhibit significantly increased Ca^2+^. Female HHR myocytes show augmented contractility with substantially lower transient Ca^2+^. **d** Phase loop analysis shows male, but not female, HHR cardiomyocytes exhibit a modified Ca^2+^-shortening relationship. **e** Male HHR cardiomyocytes exhibit significantly lower level of myofilament Ca^2+^ sensitivity, with an approximate two to threefold increase in Ca^2+^ levels at 50 % myocyte relaxation. (*sex *p* < 0.05; #strain *p* < 0.05; †sex-strain interaction; §post hoc male vs female *p* < 0.05; mean ± SEM; *n* = 5–8 cells from four to six hearts/group)

During the simulated reperfusion phase of the protocol, myocytes of all groups most typically exhibited a rapid decrease in resting length (i.e. a diastolic contracture response) with decrement in twitch amplitude, followed by gradual return to a new steady state in the extent of myocyte shortening, albeit diastolic relaxation state was only partially regained (Fig. 4a). Myocyte attrition (acute necrosis/oncosis) was observed throughout both the ischemic and reperfusions phases of the protocol (Fig. 5a). In males, there was a trend for hypertrophic myocytes (HHR) to be more resistant to cell death during the simulated ischemia/reperfusion challenge compared with normal myocytes (NHR), though this did not reach significance (Fig. 5a). A strain trend was not apparent in female myocytes. All groups showed full recovery from ischemic diastolic contracture, with resting cardiomyocyte length lower at end reperfusion compared with the pre-ischemic basal level (Fig. 5b). The extent of this reduction was greatest in male NHR cardiomyocytes and significantly greater than in female NHR cardiomyocytes. This sex difference was not apparent when cardiomyocytes were hypertrophic. During the first 5 min of reperfusion, spontaneous contractions were evident in male NHR (one of eight cardiomyocytes) and HHR (four of nine cardiomyocytes) cardiomyocytes, contrasting with a lack of spontaneous contractions in both female NHR and HHR cardiomyocytes, At the end of the 30-min simulated reperfusion challenge, surviving male and female cardiomyocytes from normal and hypertrophic hearts exhibited very different recovery parameters (Fig. 5c, †*p* < 0.05). Male HHR cardiomyocytes demonstrated equivalent shortening to male NHR controls, yet amplitude Ca^2+^ was significantly increased. This directly contrasted with female HHR cardiomyocytes, where shortening was significantly increased compared with female NHR controls, yet amplitude Ca^2+^ was not different. Phase loop analyses were performed to assess individual myocyte Ca^2+^-shortening relationships (Fig. 5d). Male, but not female, HHR cardiomyocytes exhibited a large decrease in myofilament Ca^2+^ sensitivity, with an approximate two- to threefold increase in Ca^2+^ levels at 50 % myocyte relaxation compared with male NHR and female NHR and HHR cardiomyocytes (Fig. 5e, †*p* < 0.05). These data demonstrate that, in contrast to hypertrophic male myocytes, myofilament Ca^2+^ responsiveness in hypertrophic female cardiomyocytes was not characterized by ‘desensitization’. Thus, a major sex-specific stress response phenotype difference emerges between female and male hypertrophic cardiomyocytes when subjected to simulated ischemia and reperfusion challenge.

## Discussion

This investigation provides new evidence that male and female hearts with an underlying pathological hypertrophy respond differently to cellular stresses. Using the novel HHR model (a model of normotensive intrinsic hypertrophy derived through a multigenerational program of selective breeding), our findings document pronounced cardiac and cardiomyocyte enlargement in females—including cardiomyocyte dimensional increase in female HHR equivalent to male HHR. Under basal conditions, a lower twitch amplitude in female myocytes was prominent in normal (NHR) but not in hypertrophic (HHR) myocytes. The cardiomyocyte Ca^2+^ responses to β-adrenergic challenge differed in hypertrophic male and female cardiomyocytes, with the accentuated response in males abrogated in females—even while contractile responses were similar. In simulated ischemia, the Ca^2+^ transient responses of hypertrophic male and female myocytes were markedly different—though again, all groups demonstrated similar shifts in myocyte contractile performance. A prominent and selective elevation of end-ischemia Ca^2+^ in female NHR was completely suppressed in female hypertrophic myocytes. Similarly, after the 30-min simulated reperfusion, male and female cardiomyocytes evinced different Ca^2+^ response patterns indicating an absence of the Ca^2+^ desensitization characterizing the male response.

Our data demonstrate that cardiac hypertrophy produces dramatically different basal and stress-induced pathophenotypes in female- and male-origin cardiomyocytes. The lower Ca^2+^ operational status characteristic of female (vs male) cardiomyocytes comprising normal hearts is not exhibited by myocytes of hypertrophic hearts. After ischemia/reperfusion, availability of activator Ca^2+^ is suppressed in female hypertrophic myocytes, whereas sensitivity to Ca^2+^ is blunted in male hypertrophic myocytes. These findings demonstrate that selective intervention strategies should be pursued to optimize post-ischemic electromechanical support for male and female hypertrophic hearts.

### Normal female-male cardiomyocyte contractile performance differential is eliminated with concomitant hypertrophy

The present study assessed male/female cardiomyocyte function and stress responses in a novel pathological hypertrophic HHR strain (vs control NHR strain). Significantly lower body weight in female rats is consistent with previous reports [23, 37–39], as are our findings that cardiomyocyte dimensions are smaller in normal females (vs male NHR) [24, 40–42]. This male/female cell size difference is not always evident and may be dependent on animal strain, aging/disease context and the experimental conditions under which myocyte dimensions are measured (e.g. resting Ca^2+^ concentration) [37, 43]. The possibility that there is a fundamental relationship between cardiomyocyte size and electromechanical characteristics which may partly reflect a sex-dependent size-scaling effect merits future investigation. The present finding that under standardized basal conditions, normal female cardiomyocytes contract at a lower operating level than male myocytes is consistent with our earlier report (subsequently confirmed by others) [16, 39]. This feature of female cardiomyocyte performance suggests a relatively ‘economical’ basal metabolic state. There are discrepancies within the literature regarding the mechanisms driving this female phenotype, though changes in Ca^2+^ transporter function are undoubtedly implicated. While differences in protein expression levels of various Ca^2+^ transporters have been reported between males and females in some contexts [15, 30], oestrogen regulation of the activity of some key Ca^2+^ handling effectors is likely to be of most functional importance [15, 44–48]. These previous studies assessing Ca^2+^ transporter expression/activity have utilized a series of different species (e.g. mouse, rat, guinea pig) and animal ages, which may explain variability in findings. We have recently shown that expression of the sarcoplasmic reticulum (SR) Ca^2+^ release channel (RyR2) does not differ in male/female Sprague Dawley (SpD) rats (12–16 weeks) [30], similar to that reported in 5–10-month-old C57Bl/6 mice [25]. This contrasts to previous studies in SpD and Wistar rats (age not stated), reporting an increased expression of the SR Ca^2+^ release channel (RyR2) [49, 50]. We also showed no difference in sarcoplasmic/endoplasmic reticulum Ca^2+^ ATPase (SERCA2a) and phospholamban (PLB) expression in male/female SpD rats, consistent with previous reports [49]. Directly relevant to the present study, we have shown that expression of RyR2, SERCA2a and PLB was not different in female HHR and NHR ventricular homogenates [30]. Our data indicate that it is the regulation of Ca^2+^ transporter activity, and not the expression, that differs in males/females. In particular, we have shown that basal Ca^2+^/calmodulin-dependent kinase II (CaMKII) phosphorylation (and hence, CaMKII-mediated phosphorylation of RyR2 and PLB) is lower in females, a mediator which exerts major influence on numerous Ca^2+^ transporter properties [30].

Estrogenic influence on cardiomyocyte Ca^2+^ handling includes voltage-gated Ca^2+^ current suppression, internal Ca^2+^ store release modulation and myofilament Ca^2+^ sensitivity alteration [25, 46, 51–59]. The sex-specific distinguishing characteristics in contractile phenotypes in non-hypertrophic myocytes can be partially attributed to a Ca^2+^-dependent mechanism, but likely non-Ca^2+^ dependent estrogenic actions (cell energetics/gene expression) are involved [5, 60] as the extent of contractility performance difference between males and females was more prominent than the Ca^2+^ transient amplitude differences.

### Hypertrophy is linked with accentuated response to β-adrenoceptor stimulation in male, but not female myocytes

Sex-specific aspects of altered β-adrenoceptor response in cardiac disease pathogenesis are not well characterized. Experimentally, evidence indicates that the β-adrenergic response is less pronounced in healthy cardiac tissues/myocytes of females compared with males under some circumstances. We and others have previously shown that Ca^2+^ transients are smaller in female cardiomyocytes (vs males) treated with isoproterenol [39, 61–63]. These male/female differences do not appear due to changes in β-adrenergic expression/density [64, 65], and suppressed responsiveness in females is more likely attributable to downstream signaling intermediates (i.e. adenylyl cyclase/PKA/cAMP) [25, 62].

We did not observe male/female differences in the extent of increase in twitch or Ca^2+^ transient amplitude in isoproterenol-treated NHR myocytes—suggesting that pacing under near-physiological conditions minimizes response difference [65]. Notably, male/female differences in response to isoproterenol were evident in hypertrophic myocytes. Hypertrophic female myocytes did not exhibit the accentuated elevation in Ca^2+^ transient amplitude observed in male HHR myocytes, even though both female and male HHR had similarly augmented contractile responses. This suggests a Ca^2+^-independent component determining adrenergic response selectively in females. The mechanism is unclear, but may be related to differential cellular metabolism profile—with hypertrophic hearts drawing on sex-selective energy reserves to effect an adrenergic-driven contractile response [5, 66].

### Cardiomyocyte Ca^2+^ handling and responsiveness during ischemia and reperfusion is different in hypertrophy in females and males

In this investigation, distinct mechanisms underlying vulnerability to ischemia/reperfusion in female- and male-origin cardiomyocytes are identified. These findings build on our prior observations involving isolated ex vivo hearts where we established that the more robust functional recovery of female hearts to ischemic insult was abrogated in hypertrophy [23]. In that study, HHR male and female hearts performed equally poorly, displayed similar necrotic damage (lactate dehydrogenase release) and the enhanced activation of the pro-survival reperfusion injury survival kinase (RISK; [23]) pathway seen in NHR female hearts was absent in HHR female hearts. In the two heart strains, coronary flow during ischemia/reperfusion in that study was also not different—eliminating vascular perfusion status as an underlying causative agent in sex- and strain-related performance. Our new in vitro finding presented in this investigation, that overall isolated cardiomyocyte viability loss through simulated ischemia/reperfusion was not different between sexes or strains, indicates that the measures of ex vivo heart damage reflect primarily cell rupture injury attributable to in situ tissue torsion (likely during contracture) and not cell-origin oncotic lesion.

During ischemia, female NHR showed a surprising tolerance for elevated cardiomyocyte Ca^2+^ levels—suggesting that the interplay between Ca^2+^, Na^+^ and pH which is an important determinant of electromechanical status at end reperfusion operates differently in normal male and female myocytes. Related, but qualitatively different, observations have been previously reported. The sex differences in ionic homeostatic responses to simulated ischemia may depend on precise experimental conditions [24]. Sex-comparative data relating to hypertrophic cardiomyocytes have not previously been presented. An exploration of how these ionic flux responses may be differentially manipulated via pharmacologic means may provide a valuable therapeutic lead for new post-ischemia sex- and disease-specific intervention approaches.

Cell shortening is typically reduced by approximately 50 % relative to basal in the first 5 min of simulated ischemia, though this reduction was attenuated in female HHR cardiomyocytes (Fig. 4a). This 50 % reduction is similar to that described previously [24, 67], though not as extensive as other studies describing near cessation of myocyte shortening [35, 36, 68–70]. As was observed in isolated HHR hearts [23], at the end of ischemia, male and female isolated cells showed similar levels of contractile recovery (whereas in NHR sex-specific performance, differences were apparent). Most dramatically, different underlying Ca^2+^ handling and response characteristic produced this similar net effect in HHR cell types. In the male HHR myocytes, a significant elevation of Ca^2+^ transient amplitude occurred with a major offsetting reduction in myofilament sensitivity to Ca^2+^. In the female HHR myocytes, this combination of Ca^2+^ adaptations was not apparent. In relation to adverse outcomes in early reperfusion, these findings may be extrapolated to suggest that in acute reperfusion, Ca^2+^ overload arrhythmogenic predisposition presents as a male myocyte vulnerability. In contrast, the blunted female myocyte Ca^2+^ transient response to β-adrenoceptor inotropic influence has the potential for compromised functional performance. Thus, ischemic recovery is similarly impaired in both males and females—but via different cellular mechanisms. Clinically, the importance of gender differences in arrhythmia substrate is increasingly recognized—and advancing the understanding of underlying cellular processes involved is essential [71].

The present study, of young adult rodents at prime estrogenic life stage, demonstrates the absence of a female ‘protected’ state in the context of hypertrophy. The conventional view of female oestrogen cardioprotection may require refinement [15, 72]. Oestrogen receptors (ERα, oestrogen receptor β (ERβ), G-protein-coupled oestrogen receptor (GPER)) are expressed in male and female myocardium. When expression/activity is manipulated genetically or pharmacologically, dual sex effects may be observed [73]—yet oestrogen exposure is not always favourable [15]. Experimentally, where female hearts have shown more ischemic resilience than male hearts, this has been most evident when the context involved baseline elevated Ca^2+^ loading levels [20–22]. New information suggests that local myocardial androgen-to-oestrogen conversion may be an element in determining the sex specificity of responses to cardiac challenge [74–76], and in hypertrophy, local steroid levels may become more important in modulating function under stress conditions. More detailed studies of the specific molecular processes involved in regulating ionic homeostasis in male- and female-origin cardiomyocytes in defined disease hypertrophic states and acute stress settings are warranted. A limitation of the present study is the in vitro and ‘unloaded’ conditions under which myocyte recordings are undertaken. However, the advantage of the approach is the capacity to achieve a high level of control over cellular ambience to resolve key functional differences.

## Conclusions

In summary, this experimental investigation demonstrates that the responses of hypertrophic male and female cardiomyocytes to β-adrenoceptor stimulation and ischemia/reperfusion challenges are fundamentally different at the cellular level—and that hypertrophy in females confers a distinctive liability. Our findings indicate that therapeutic interventions targeted to the early reperfusion period may have differential sex-specific efficacy depending on the presence of an underlying comorbid hypertrophic state. Where clinical trials have previously failed to identify benefit in early reperfusion manipulation of cellular processes impacting on myofilament Ca^2+^ sensitivity, there may be a case for review in selected at-risk female subgroups. This study provides a basis to support relevant focused pre-clinical research to pursue this possibility.

## Abbreviations

ANOVA, analysis of variance; CaMKII, Ca^2+^/calmodulin-dependent kinase II; CWI, cardiac weight index; ERα, oestrogen receptor α; ERβ, oestrogen receptor β; GPER, G-protein-coupled oestrogen receptor; HEPES, (4-(2-hydroxyethyl)-1-piperazineethanesulfonic acid; HHR, hypertrophic heart rat; NHR, normal heart rat; RISK, reperfusion injury salvage kinase; SHR, spontaneously hypertensive rat

## Additional files

Additional file 1: Table S1. Basal sex differences in cardiomyocyte performance suppressed with hypertrophy. (*sex *p* < 0.05, #strain *p* < 0.05; mean ± SEM, *n* = hearts or animals in brackets for each group). (PPTX 68 kb) Additional file 2: Table S2. Sex differences in cardiomyocyte performance following either β-adrenoceptor stimulation or stimulated ischemia. (Values expressed as % basal. *sex *p* < 0.05, # strain *p* < 0.05; mean ± SEM, *n* = cells in brackets for each group). (PPTX 62 kb)
