# Supplementary material for: Male and female hypertrophic rat cardiac myocyte functional responses to ischemic stress and β-adrenergic challenge are different
Source: Biol Sex Differ. 2016 Jul 7;7:32. doi: 10.1186/s13293-016-0084-8 (PMC4936311; doi:10.1186/s13293-016-0084-8)
Supplement: Additional file 1: Table S1. — Basal sex differences in cardiomyocyte performance suppressed with hypertrophy. (*sex p < 0.05, #strain p < 0.05; mean ± SEM, n = hearts or animals in brackets for each group). (PPTX 68 kb) [file 13293_2016_84_MOESM1_ESM.pptx]

## Slide 1
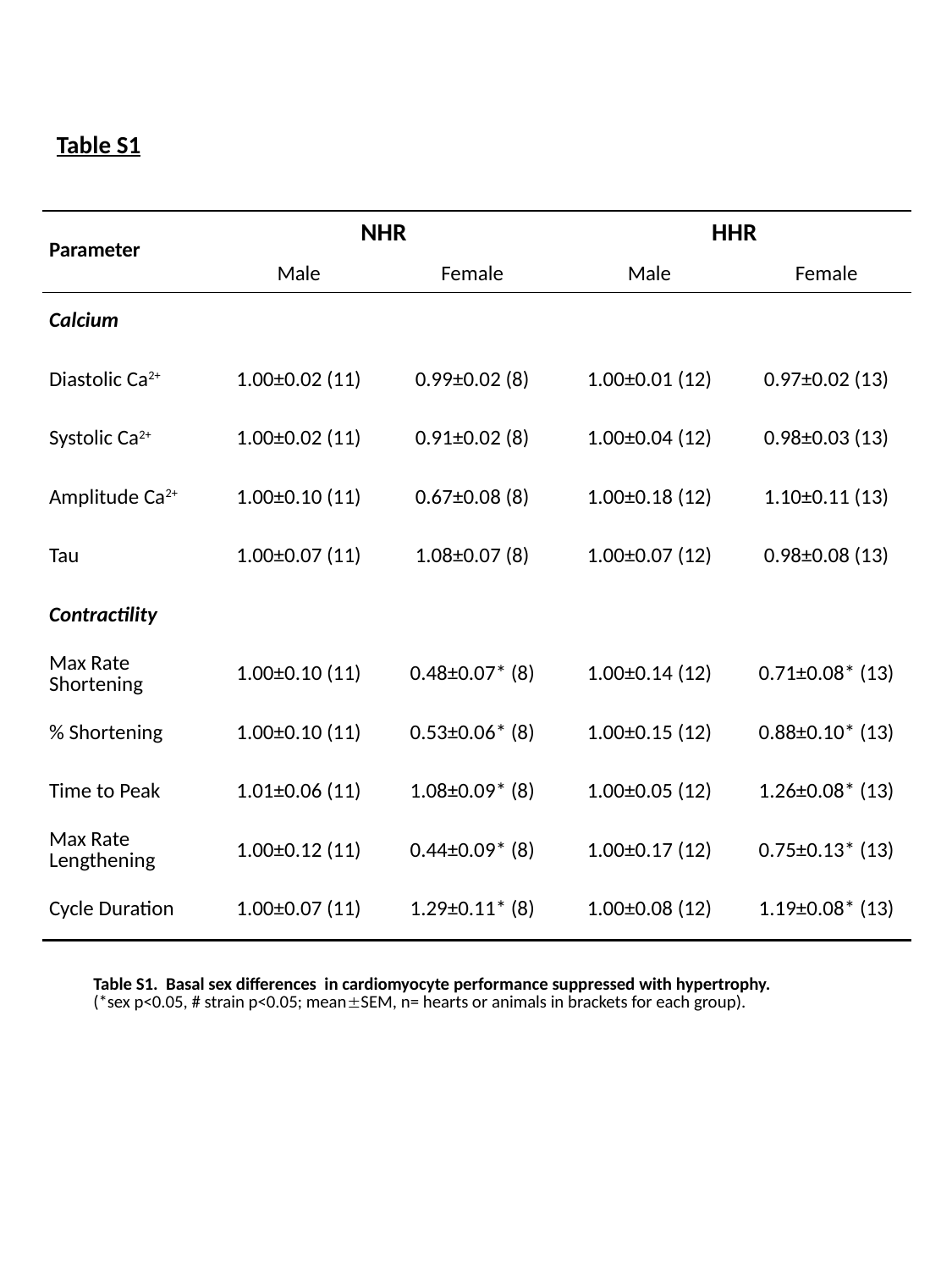

Table S1
| Parameter | NHR | | HHR | |
| --- | --- | --- | --- | --- |
| | Male | Female | Male | Female |
| Calcium | | | | |
| Diastolic Ca2+ | 1.00±0.02 (11) | 0.99±0.02 (8) | 1.00±0.01 (12) | 0.97±0.02 (13) |
| Systolic Ca2+ | 1.00±0.02 (11) | 0.91±0.02 (8) | 1.00±0.04 (12) | 0.98±0.03 (13) |
| Amplitude Ca2+ | 1.00±0.10 (11) | 0.67±0.08 (8) | 1.00±0.18 (12) | 1.10±0.11 (13) |
| Tau | 1.00±0.07 (11) | 1.08±0.07 (8) | 1.00±0.07 (12) | 0.98±0.08 (13) |
| Contractility | | | | |
| Max Rate Shortening | 1.00±0.10 (11) | 0.48±0.07\* (8) | 1.00±0.14 (12) | 0.71±0.08\* (13) |
| % Shortening | 1.00±0.10 (11) | 0.53±0.06\* (8) | 1.00±0.15 (12) | 0.88±0.10\* (13) |
| Time to Peak | 1.01±0.06 (11) | 1.08±0.09\* (8) | 1.00±0.05 (12) | 1.26±0.08\* (13) |
| Max Rate Lengthening | 1.00±0.12 (11) | 0.44±0.09\* (8) | 1.00±0.17 (12) | 0.75±0.13\* (13) |
| Cycle Duration | 1.00±0.07 (11) | 1.29±0.11\* (8) | 1.00±0.08 (12) | 1.19±0.08\* (13) |
Table S1. Basal sex differences in cardiomyocyte performance suppressed with hypertrophy.
(*sex p<0.05, # strain p<0.05; mean±SEM, n= hearts or animals in brackets for each group).
