# Supplementary material for: Male and female hypertrophic rat cardiac myocyte functional responses to ischemic stress and β-adrenergic challenge are different
Source: Biol Sex Differ. 2016 Jul 7;7:32. doi: 10.1186/s13293-016-0084-8 (PMC4936311; doi:10.1186/s13293-016-0084-8)
Supplement: Additional file 2: Table S2. — Sex differences in cardiomyocyte performance following either β-adrenoceptor stimulation or stimulated ischemia. (Values expressed as % basal. *sex p < 0.05, # strain p < 0.05; mean ± SEM, n = cells in brackets for each group). (PPTX 62 kb) [file 13293_2016_84_MOESM2_ESM.pptx]

## Slide 1
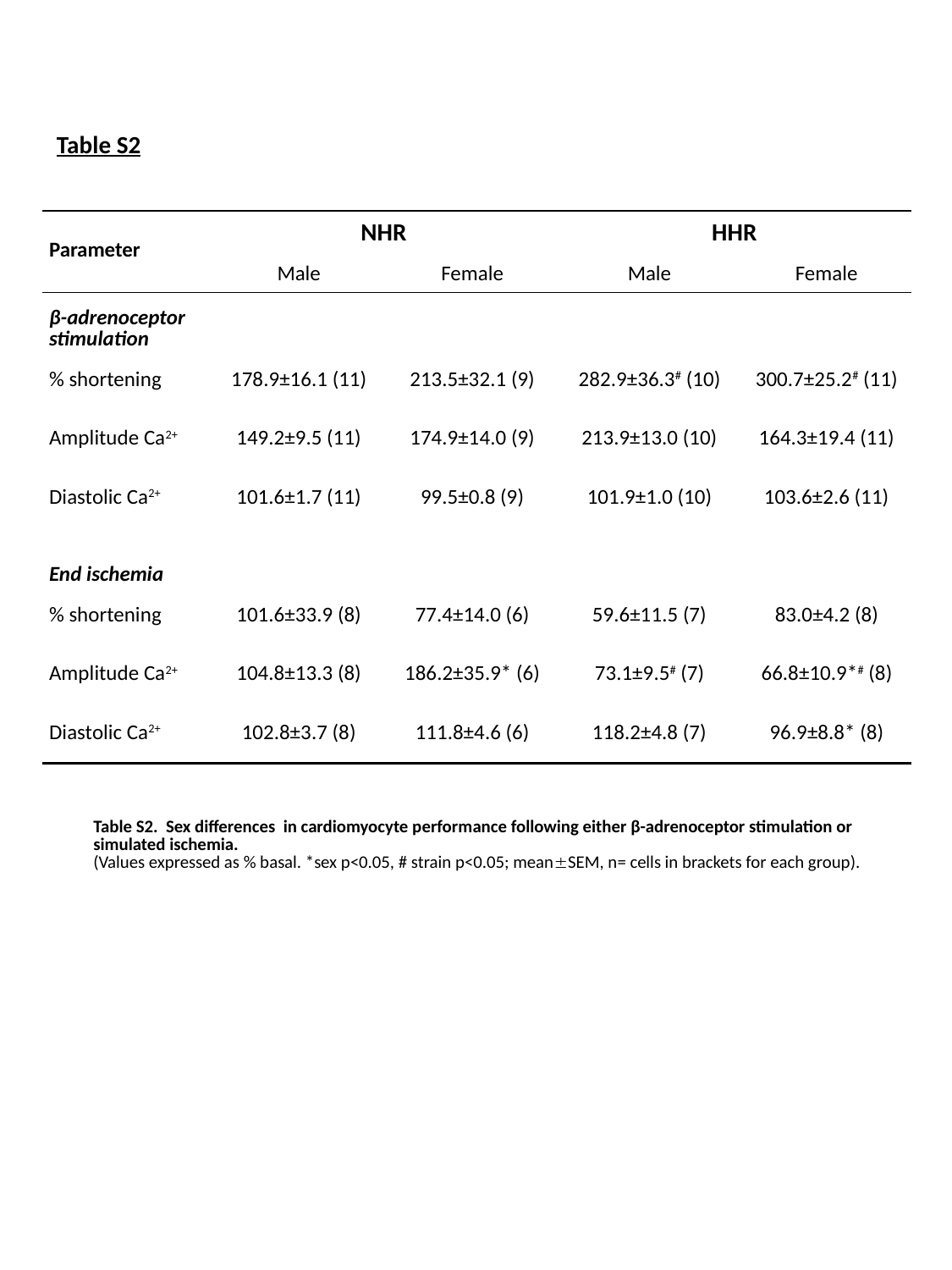

Table S2
| Parameter | NHR | | HHR | |
| --- | --- | --- | --- | --- |
| | Male | Female | Male | Female |
| β-adrenoceptor stimulation | | | | |
| % shortening | 178.9±16.1 (11) | 213.5±32.1 (9) | 282.9±36.3# (10) | 300.7±25.2# (11) |
| Amplitude Ca2+ | 149.2±9.5 (11) | 174.9±14.0 (9) | 213.9±13.0 (10) | 164.3±19.4 (11) |
| Diastolic Ca2+ | 101.6±1.7 (11) | 99.5±0.8 (9) | 101.9±1.0 (10) | 103.6±2.6 (11) |
| End ischemia | | | | |
| % shortening | 101.6±33.9 (8) | 77.4±14.0 (6) | 59.6±11.5 (7) | 83.0±4.2 (8) |
| Amplitude Ca2+ | 104.8±13.3 (8) | 186.2±35.9\* (6) | 73.1±9.5# (7) | 66.8±10.9\*# (8) |
| Diastolic Ca2+ | 102.8±3.7 (8) | 111.8±4.6 (6) | 118.2±4.8 (7) | 96.9±8.8\* (8) |
Table S2. Sex differences in cardiomyocyte performance following either β-adrenoceptor stimulation or simulated ischemia.
(Values expressed as % basal. *sex p<0.05, # strain p<0.05; mean±SEM, n= cells in brackets for each group).
